# Choroidal spatial distribution indexes as novel parameters for topographic features of the choroid

**DOI:** 10.1038/s41598-019-57211-2

**Published:** 2020-01-17

**Authors:** Sungsoon Hwang, Mingui Kong, Yun-Mi Song, Don-Il Ham

**Affiliations:** 1 Department of Ophthalmology, Samsung Medical Center, Sungkyunkwan University School of Medicine, Seoul, Republic of Korea; 2 Hangil Eye Hospital, Incheon, Republic of Korea; 3 Department of Ophthalmology, Catholic Kwandong University College of Medicine, Incheon, Republic of Korea; 4 Department of Family Medicine, Samsung Medical Center, Sungkyunkwan University School of Medicine, Seoul, Republic of Korea

**Keywords:** Retina, Eye manifestations

## Abstract

The purpose of the study is to propose choroidal spatial distribution indexes (CSDIs) to represent choroidal topographic features, establish a normative database for CSDIs, and identify factors associated with CSDIs in healthy eyes. Retrospective data analysis of 363 healthy eyes from a single-center, prospective, cross-sectional, non-interventional study. Subjects were evaluated using spectral domain OCT with enhanced depth imaging. Choroidal volume and average thickness were measured with OCT in nine macular subfields defined by the Early Treatment Diabetic Retinopathy Study. Vertical CSDI was defined as the natural logarithm of superior choroidal volume divided by inferior choroidal volume. Horizontal CSDI was defined as the natural logarithm of temporal choroidal volume divided by nasal choroidal volume. The overall distributions of vertical and horizontal CSDIs was analyzed. Linear regression analyses were used to identify ocular and systemic factors associated with vertical and horizontal CSDIs. The average vertical CSDI was 0.062 ± 0.206, and average horizontal CSDI was 0.138 ± 0.226. Both vertical and horizontal CSDIs followed normal distribution. Increasing age was significantly associated with greater vertical CSDI (choroidal volume distribution tilted toward the superior region), and longer axial length and thinner subfoveal choroidal thickness were significantly associated with greater horizontal CSDI (choroidal volume distribution tilted toward the temporal region).

## Introduction

The choroid is the vascular layer of the eye with the highest blood flow of any tissue in the human body^1^. As a major vascular layer, the choroid plays an important role in ocular health and is involved in a number of ocular diseases such as central serous chorioretinopathy, age-related macular degeneration, polypoidal choroidal vasculopathy, and myopic macular degeneration^2–6^. Thus, there needs to be an emphasis on gaining a better understanding of choroidal structure.

Recently, the advent of enhanced depth imaging optical coherence tomography (OCT) and swept-source OCT has provided more precise and detailed assessment of the choroid^7–9^. Along with the advancement of imaging technology, choroid assessment in the literature has shifted from merely measuring subfoveal choroidal thickness^2,5,6^ to calculating the entire choroidal volume. The latter is done by using the Early Treatment Diabetic Retinopathy Study (ETDRS) grid^10–12^ as measuring choroidal thickness from a single or few sampling points can be easily influenced by local changes in choroidal thickness or irregularities in the choroidoscleral border ^4,5,13,14^.

Volume scanning of the choroid has led to deeper understanding of topographical features of the choroid. The spatial distribution of the choroid was found to be more complex and varied compared to that of the retina. Choroidal distribution is not necessarily centered at the fovea; it can be displaced in either direction of the macula, and the amount and direction of displacement varies between individuals^10,12,15–17^. Though there have been many studies reporting on the topographic features of choroidal volume, as yet, there are no common markers or numerical indexes to describe spatial distribution of choroidal volume. To evaluate spatial distribution, previous studies have only subtracted and directly compared choroidal volume or thickness of specific ETDRS subfields^10,12^.

In the current study, we propose new parameters called choroidal spatial distribution indexes (CSDIs) to quantify the topographic distribution of the choroid. Furthermore, we present the distribution of CSDIs in healthy eyes, and determine the ocular and systemic factors affecting CSDIs based on a population-based twin and family study done in South Korea. This index may provide an easy and efficient way to demonstrate spatial features of the choroid in a quantitative fashion.

## Methods

### The healthy twin study

The data for this study were derived from the Healthy Twin Study, a nationwide population-based study initiated in 2005. The Healthy Twin Study recruited Korean adult twins and their family members to investigate genetic and environmental determinants of a wide range of traits. The study adhered to the tenets of the Declaration of Helsinki and was approved by the Institutional Review Board of Samsung Medical Center, Seoul, Republic of Korea. Written informed consent was obtained from all participants after study details, and potential risks and consequences were explained. A more detailed description of the methodology and protocols of the Healthy Twin Study have been reported elsewhere^18,19^.

### Study subjects

The current study included 363 participants from the Healthy Twin Study who had undergone a macular volumetric OCT scan in the Department of Ophthalmology at Samsung Medical Center, Seoul, South Korea. Exclusion criteria for subjects included those who had a history of ocular surgery or ocular diseases that might have affected retinal and choroidal thicknesses (i.e., severe cataract, glaucoma, epiretinal membrane, diabetic retinopathy, retinal vein occlusion, age-related macular degeneration, and pathologic myopia) and those whose OCT images were of inadequate quality and inappropriate for evaluation of choroidal volume.

### Choroidal volume assessment

Macular volumetric OCT scans were obtained using the Spectralis HRA + OCT (version 1.7.0.0; Heidelberg Engineering, Heidelberg, Germany) without pupil dilation. All OCT scans were operated by a single well-trained technician. The raster scan image was composed of 31 B-scans, each consisting of 768 A-scans, 9.0 mm in length, spaced 240 μm apart, and covering a 30-degree × 25-degree area. Automatic real-time mode using the eye tracker system was activated, with a total of 25 frames averaged for one B-scan image. As recommended by the manufacturer instructions, participants’ keratometry and refraction values were entered into the software program to estimate optical magnification. No manual correction was applied to the OCT output. Images were required to have a quality index of at least 20 to be included in the study and any images with artifacts were excluded.

We selected the retinal thickness map analysis protocol to display the volume and average thickness of the retinal layer for each of the nine subfields defined by the ETDRS (central, four inner quadrants, and four outer quadrants)^20^. For assessment of choroidal volume, segmentation lines in all 31 horizontal scans of each participant were manually changed; the internal limiting membrane layer was moved to the outer part of the retinal pigment epithelium (RPE) level, and the Bruch’s membrane segmentation line was moved to the outer border of the choroid^21^. We obtained the choroidal volume and numerical averages of choroidal thickness for each of the nine subfields displayed using the thickness map analysis software. Segmentation was performed by two retinal specialists (S.H. and M.K.) who were masked to each other’s measurements. The inter-observer reproducibility was assessed between the two acquired datasets, and the mean values of the two measurements were used for the subsequent analyses in this study.

### Definition of choroidal spatial distribution index

CSDIs were composed of vertical and horizontal indexes, defined as follows:

$${\rm{vertical}}\,{\rm{CSDI}}=\,\mathrm{ln}(\frac{superior\,choroidal\,volume}{inferior\,choroidal\,volume})$$

$${\rm{horizontal}}\,{\rm{CSDI}}=\,\mathrm{ln}(\frac{temporal\,choroidal\,volume}{nasal\,choroidal\,volume})$$

**Figure 1:**
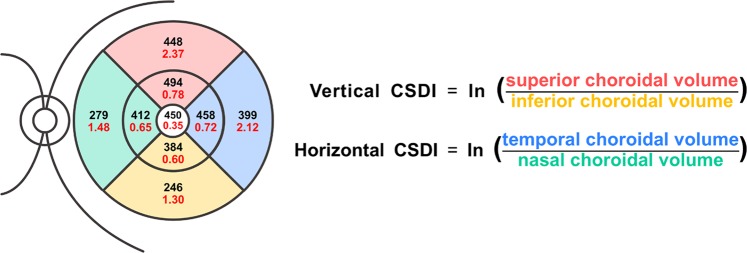
Calculation of vertical and horizontal choroidal spatial distribution index (CSDI). Black numbers and red numbers in each subfield represent mean choroidal thickness (μm) and choroidal volume (mm^3^) of corresponding subfield, respectively. Vertical CSDI is the natural logarithm (ln) of the superior choroidal volume divided by the inferior choroidal volume. Horizontal CSDI is the natural logarithm of the temporal choroidal volume divided by the nasal choroidal volume. Superior choroidal volume is the sum of the superior inner and outer subfield (red shade) choroidal volumes, and inferior choroidal volume is the sum of the inferior inner and outer subfield (yellow shade) choroidal volumes. Temporal choroidal volume is the sum of the temporal inner and outer subfield (blue shade) choroidal volumes, and nasal choroidal volume is the sum of the nasal inner and outer subfield (green shade) choroidal volumes.

**Figure 2:**
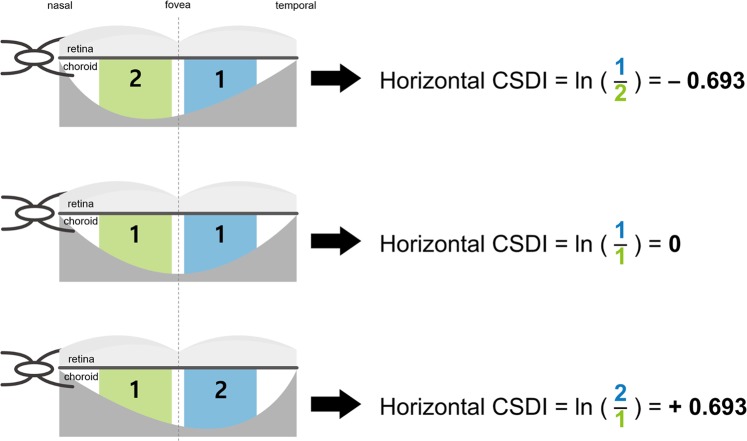
Horizontal choroidal spatial distribution index (CSDI) in nasally tilted, symmetrical, and temporally tilted choroidal distribution. When nasal choroidal volume is twice that of temporal choroidal volume, horizontal CSDI is −0.693. If nasal choroidal volume and temporal choroidal volume are equal, horizontal CSDI is 0. When nasal choroidal volume is half that of temporal choroidal volume, horizontal CSDI is +0.693. Horizontal CSDI has a negative value when the choroid is tilted nasally, zero when the choroid is symmetrically distributed around the fovea, and has a positive value when the choroid is tilted temporally. ln: natural logarithm.

**Figure 3:**
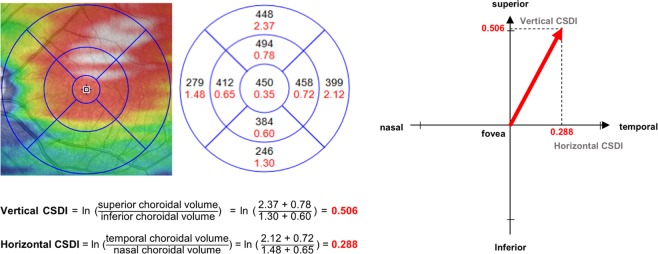
Interpretation of both vertical and horizontal choroidal spatial distribution indexes (CSDI) for choroidal spatial distribution. (Left) Eyes with a choroidal distribution inclined toward the superotemporal area show positive vertical and horizontal CSDIs. (Right) By obtaining vertical and horizontal CSDIs, we can determine the direction and degree bias of the choroidal spatial distribution from the fovea. ln: natural logarithm.

Vertical CSDI indicates how much the choroidal volume is tilted toward the superior region and horizontal CSDI indicates how much the choroidal volume is tilted toward the temporal area, both using the fovea as the reference point. Figure 1 outlines how to calculate vertical and horizontal CSDIs from the display of the Spectralis software. Superior choroidal volume is the sum of the choroidal volume of the superior inner and outer subfields, and inferior choroidal volume is the sum of the choroidal volume of the inferior inner and outer subfields. The same calculation is applied for the temporal and nasal choroidal volumes. Figure 2 demonstrates numerical value of horizontal CSDI in nasally tilted, symmetrical, and temporally tilted choroidal distributions; horizontal CSDI would have a negative value if the choroid is tilted nasally, would be equal to zero if the choroid is symmetrically distributed around the fovea, and would have a positive value if the choroid is tilted temporally. By obtaining the vertical and horizontal CSDIs, we can figure the degree and directional tilt of choroidal distribution. For instance, if both vertical and horizontal CSDIs have positive values, we can conclude that the choroid lies superotemporally from the fovea (Fig. 3). If both vertical and horizontal CSDIs are close to zero, it indicates that the choroid is distributed symmetrically around the fovea.

### Measurement of ocular factors

All study subjects received a comprehensive ophthalmic examination including a visual acuity assessment, measurements of intraocular pressure (IOP) and non-dilated refraction using an autorefractor (Topcon AT; Topcon Corp., Tokyo, Japan), and axial length using corneal touch A-scan ultrasonography (Model 820; Allergan-Humphrey, San Leandro, CA, USA). Color fundus photographs were also taken using a fundus camera TRC 50 (Topcon, Paramus, NJ, USA) or Nonmyd 7 (Kowa, Tokyo, Japan) to identify any retinal pathology following pupil dilation. Two retinal specialists (S.H. and M.K.) evaluated the clinical records, ocular measurements, and color fundus photographs to exclude those with a previous history of ocular surgery, decreased visual acuity in the absence of any pathological cause (amblyopia), or retinal/choroidal pathologies that could affect choroidal volume and distribution.

### Measurement of systemic factors

All study subjects underwent a routine lab blood test and blood pressure measurement. The concentrations of glucose, hemoglobin A1c, high density lipoprotein cholesterol (HDL-C), and low density lipoprotein cholesterol (LDL-C) were measured using commercially available enzymatic or homogeneous assay kits with the ADVIA 1650 analyzer (Siemens, Munich, Germany), using fresh serum collected after a minimum 12-hour overnight fast. A trained research nurse repeatedly measured blood pressure (BP) using a standard mercury sphygmomanometer and the average value of the two measurements was used for analyses. We defined hypertension as having high systolic BP (≥140 mmHg), high diastolic BP (≥90 mmHg), or current use of a BP-lowering agent. We defined diabetes as having a fasting glucose level of ≥126 mg/dL, hemoglobin A1c level of ≥6.5%, or use of a glucose-lowering agent. Weight (kg) and height (m) were measured in light clothing using standardized scales and stadiometers, and body mass index was calculated by dividing the weight by the height squared (kg/m^2^). Data on past medical history and smoking status were collected using a self-administered questionnaire. We categorized smoking status into two groups, current smoker and past/non-smoker.

### Statistical methods

Data from the right eye of every participant were used for the analyses. Statistical analysis was performed using SPSS software version 23.0 (SPSS, Inc., Chicago, IL, USA). For the assessment of inter-observer reproducibility of choroidal segmentation, intraclass correlation coefficient (ICC, two-way random effects/absolute agreement), 95% limits of agreement, and coefficient of reproducibility of the average choroidal thickness (choroidal volume divided by the area of corresponding subfield) in each of the nine macular subfields defined by the ETDRS was calculated. The distribution of the vertical and horizontal CSDIs for all participants were described using a histogram plot. A normality test was conducted for the aforementioned parameters. Univariate and multiple linear regression analyses were performed to determine the association of CSDIs with other ocular and systemic factors that could possibly affect choroidal distribution. Parameters presenting association (*P* < 0.10) in univariate analysis were included in multiple linear regression analyses. All *P* values were two-sided and considered statistically significant for values less than 0.05.

## Results

A total of 416 subjects were recruited for the study of which 53 subjects were excluded for having previous ocular surgery, amblyopia, or demonstrating retinal/choroidal pathology (n = 36), and having poor OCT image quality (n = 17). Thus, a total of 363 healthy eyes from 363 subjects were included in the analysis.

The ICC of the average choroidal thickness measured by two different observers ranged from 0.988 at the outer superior subfield to 0.995 at the inner nasal subfield. The coefficient of reproducibility ranged from 17.47 μm to 24.28 μm. The more detailed information is available in the Supplementary Table S1.

The average age was 48.5 ± 13.8 years and 141 subjects (38.8%) were male (Table 1). The average vertical CSDI was 0.062 ± 0.206 (range, −0.502–0.784) and average horizontal CSDI was 0.138 ± 0.226 (range, −0.573–0.972). The detailed demographics, ocular, systemic, and choroidal features are shown in Table 1. A one sample t-test on the vertical and horizontal CSDIs presented a *P* value of less than 0.001 for both parameters (test value = 0).

**Table 1:** Demographics, clinical and choroidal parameters of the study subjects (n = 363).

	Mean ± Standard deviation	Range
Age, years	48.5 ± 13.8	18–80
Sex, male (%)	141 (38.8%)	
Axial length, mm	23.68 ± 1.03	20.86–26.78
IOP, mmHg	14.4 ± 2.8	8.8–24.2
Hypertension (%)	63 (17.6%)	
Diabetes (%)	20 (5.5%)	
HDL-C, mg/dL	53.6 ± 11.9	29–105
LDL-C, mg/dL	193.8 ± 35.3	42–217
Body mass index, kg/m^2^	23.7 ± 3.1	16.3–34.3
Alcohol consumption (%)	214 (59.0%)	
Current smoker (%)	104 (28.7%)	
**Choroidal parameters**		
Subfoveal thickness (μm)	296.6 ± 97.2	71.5–557.5
Vertical CSDI	0.062 ± 0.206	−0.502–0.784
Horizontal CSDI	0.138 ± 0.226	−0.573–0.972

Continuous variables are described as mean ± standard deviation, and categorical parameters are described as total numbers (percentage).

IOP = intraocular pressure; HDL-C = high density lipoprotein cholesterol; LDL-C = low density lipoprotein cholesterol; CSDI = choroidal spatial distribution index.

**Figure 4:**
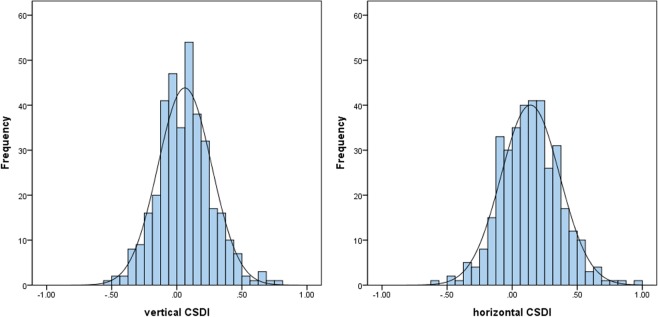
Distribution of vertical and horizontal choroidal spatial distribution indexes (CSDI) across the population. Vertical and horizontal CSDIs follow a normal distribution.

The histogram plot (Fig. 4) represents the distribution of vertical and horizontal CSDIs. Both vertical and horizontal CSDI followed a normal distribution. The Kolmogorov-Smirnov test presented a *P* value of 0.71 for vertical CSDI and 0.91 for horizontal CSDI. The Shapiro-Wilk test presented a *P* value of 0.082 for vertical CSDI and 0.49 for horizontal CSDI.

Table 2 shows the univariate and subsequent multivariate regression analysis for vertical and horizontal CSDIs. The models show increasing age to be significantly (*P* = 0.019) associated with greater vertical CSDI (tilted superiorly), and longer axial length to be significantly (*P* < 0.001) and thinner subfoveal choroidal thickness (*P* < 0.001) associated with greater horizontal CSDI (tilted temporally). No other factors were presenting statistically significant association with vertical or horizontal CSDI.

**Table 2:** Linear regression analyses of systemic and ocular factors associated with vertical and horizontal choroidal spatial distribution indexes.

	Univariate		Multivariate*	
	Standardized beta	*P* value	Standardized beta	*P* value
**Vertical CSDI**				
Age	0.164	0.002	0.129	0.019
Sex	−0.030	0.57		
Axial length	0.057	0.28		
IOP	−0.036	0.50		
Subfoveal choroidal thickness	−0.146	0.005	−0.104	0.060
Hypertension	0.032	0.54		
Diabetes	−0.017	0.75		
HDL-C	0.024	0.65		
LDL-C	−0.044	0.41		
Body mass index	−0.080	0.13		
Alcohol consumption	−0.018	0.73		
Current smoker	−0.040	0.45		
**Horizontal CSDI**				
Age	−0.108	0.040	−0.105	0.063
Sex	0.017	0.744		
Axial length	0.284	<0.001	0.215	<0.001
IOP	0.046	0.38		
Subfoveal choroidal thickness	−0.266	<0.001	−0.283	<0.001
Hypertension	−0.006	0.91		
Diabetes	−0.111	0.034	−0.076	0.133
HDL-C	0.081	0.12		
LDL-C	−0.114	0.030	−0.078	0.124
Body mass index	−0.116	0.027	−0.096	0.060
Alcohol consumption	−0.032	0.55		
Current smoker	0.015	0.78		

^*^Adjusted for variables with a *P* value < 0.10 in the univariate analysis.

IOP = intraocular pressure; HDL-C = high density lipoprotein cholesterol; LDL-C = low density lipoprotein cholesterol; CSDI = choroidal spatial distribution index.

## Discussion

In this population-based cross-sectional study, we propose CSDIs to represent topographic features of the choroid. CSDIs are intuitively understandable parameters designating the degree of and direction in which the overall choroidal volume is inclined and can be easily calculated based on choroidal volume measured by OCT. Given that volume scanning of the choroid has recently been automated and widely used in numerous studies, these new spatial indexes may be employed in various studies investigating choroidal distribution and may help further elucidate the role of the choroid in disease development and progression.

There have been several previous studies exploring the spatial distribution of choroidal thickness and volume as measured by OCT; however, the methods used for topographic analysis differed between studies. Ouyang *et al*. directly compared the value of average choroidal thickness in all nine ETDRS subfields^10^. Sanchez-Cano *et al*. subtracted the choroidal thicknesses and volumes in each of the ETDRS areas and obtained differences between mean choroidal thicknesses and volumes in different areas to obtain topographic information^12^. Lee *et al*. used a ratio of temporal and nasal choroidal thickness to foveal choroidal thickness to describe topographical variation of macular choroidal thickness in myopic patients^22^. The methodological disparity between these studies may have led to inconsistent and discordant interpretation of the data; this necessitates uniform indicators for the study of choroidal volume distribution.

Our study revealed that, on average, both vertical and horizontal CSDIs are greater than zero, i.e., in general, choroidal volume distribution from the fovea was tilted toward the superior and temporal areas. Previous studies have shown choroidal thickness to be greatest at the fovea with decreasing thickness more nasally than temporally^8,23^. Others have suggested choroidal thickness to be greatest in the regions temporal and superior to the fovea, not the fovea itself. For instance, Ouyang *et al*. and Hirata *et al*. suggested that, on average, the choroid is thickest in the superior outer ETDRS subfield and thinnest in the nasal outer ETDRS subfield^9,10^. Further, Sanchez-Cano *et al*. suggested that the choroid is thickest in the superior and temporal areas^12^. Our study noted that, on average, vertical and horizontal CSDIs had positive values, in accordance with these results.

In addition, we also presented that increasing age was significantly associated with greater vertical CSDI (choroidal volume distribution tilted superiorly), and a longer axial length and thinner subfoveal choroidal thickness was significantly associated with greater horizontal CSDI (choroidal volume distribution tilted temporally). The link between axial length and temporalization of choroidal distribution has been reported in many previous studies. Fujiwara *et al*. reported that the temporal choroid was thicker than the subfoveal choroid in highly myopic eyes^6^. Lee *et al*. also showed that elongation of the globe was highly associated with relative temporal choroidal thickening^22^. A hypothesis for this phenomenon was proposed by Chui *et al*., who suggested that during axial elongation, the scleral and choroidal layers may stretch in the temporal direction, with relatively less stretch exhibited by the retinal layer as it is essentially part of the nervous system^24^. As of interest, thinner choroidal thickness was also associated with temporal tilting of choroidal distribution even after the adjustment of axial length. It seems to have directionality when the choroidal thickness changes. The choroidal thickness should be considered while interpreting the spatial distribution of choroid. With regards to vertical CSDI, Ouyang *et al*. reported that increasing age was associated with more dramatic choroidal thickness decreases in the inferior macula versus those seen superiorly, which is similar to that noted in our study^10^. However, in contrast to the association between axial length elongation and temporalization of choroidal distribution, there is as yet no plausible explanation for the association between increasing age and superior tilting of the choroidal volume. Further investigation is required to develop a possible hypothesis for this association.

The current study had several strengths. First, it included a large number of participants, which allowed us to achieve a high statistical power and validate the distribution of these newly proposed spatial indexes. To the best of our knowledge, this is the largest study of choroidal volume evaluation performed manually. Second, the study was carried out in a single ethnic population, therefore unlikely to be influenced by ethnic heterogeneity. Third, the study used standardized clinical examination protocols and only included healthy eyes so that measurement errors were minimized. Also, the OCT was performed without pupil dilation in this study. Since pupil dilation using phenylephrine drops is reported to thin the choroidal tissue^25^, our study is free from the measurement error which could be induced by pupil. Lastly, the reproducibility of manual choroidal segmentation was very high in our study (0.988–0.995) and was comparable to the previous reports^21,26^. This supports the accurate measurement of the choroidal volume in the current study.

Nevertheless, this study does have certain limitations. First, CSDIs simplify the topographic features of the choroid; they only represent tilting of the volume distribution and do not describe additional details of choroidal topography. Thus, relying solely on CSDIs to understand choroidal spatial distribution should be avoided. Second, choroidal volume was measured manually. The measurements were performed by moving the built-in reference lines of the retinal boundary to the choroidal boundary. Since this was not an automated procedure, measurement errors could have occurred. Future studies using advanced OCT instruments with reliable automated choroidal segmentation software to validate these parameters is required. Third, the OCT examinations for individual subjects were not performed at the same times of day. Since there is evidence for the diurnal variation of choroidal thickness in healthy eyes^27^, this could have affected the accuracy of the choroidal volume assessment. Fourth, the OCT was performed after pupil dilation in all participants. Pupil dilation using phenylephrine drops is reported to thin the choroidal tissue. Therefore, the current study might have underestimated the choroidal thickness.

In conclusion, we propose novel OCT-based indicators, termed CSDIs, to represent the spatial distribution of the choroid. Our results have shown that, in general in healthy eyes, the choroid is inclined superiorly and temporally and has a tendency to be tilted superiorly with increasing age and temporally with increasing axial length, respectively. These indexes could be used as a new, easily applicable method of studying the physiology of human choroidal distribution.

## Supplementary information

Supplementary Information 

## Data Availability

The datasets generated during and/or analyzed during the current study are available from the corresponding author on reasonable request.
