# Supplementary material for: Choroidal spatial distribution indexes as novel parameters for topographic features of the choroid
Source: Sci Rep. 2020 Jan 17;10:574. doi: 10.1038/s41598-019-57211-2 (PMC6969017; doi:10.1038/s41598-019-57211-2)
Supplement: Supplementary file 1 — Supplementary Information [file 41598_2019_57211_MOESM1_ESM.docx]

**Choroidal spatial distribution indexes as novel parameters for topographic features of the choroid**

Sungsoon Hwang, MD^1*^, Mingui Kong, MD^2,3*^, Yun-Mi Song, MD, PhD^4†^, Don-Il Ham, MD, PhD^1†^

^*^Sungsoon Hwang and Mingui Kong equally contributed to the manuscript as first authors.

^†^Don-Il Ham and Yun-Mi Song equally contributed to the manuscript as corresponding authors.

^1^Department of Ophthalmology, Samsung Medical Center, Sungkyunkwan University School of Medicine, Seoul, Republic of Korea

^2^Hangil Eye Hospital, Incheon, Republic of Korea

^3^Department of Ophthalmology, Catholic Kwandong University College of Medicine, Incheon, Republic of Korea

^4^Department of Family Medicine, Samsung Medical Center, Sungkyunkwan University School of Medicine, Seoul, Republic of Korea

Don-Il Ham, MD, PhD

Department of Ophthalmology, Samsung Medical Center, Sungkyunkwan University School of Medicine, #81 Irwon-ro, Gangnam-gu, Seoul 06351, South Korea

Tel: (+82)-2-3410-3548, Fax: (+82)-2-3410-0074

E-mail: oculus@naver.com

Yun-Mi Song, MD, PhD

Department of Family Medicine, Samsung Medical Center, Sungkyunkwan University School of Medicine, #81 Irwon-ro, Gangnam-gu, Seoul 06351, South Korea

E-mail: yunmisong@skku.edu

**Supplementary Table S1.** Measures of reproducibility between two observers for the average choroidal thickness in nine macular subfields.

| Subfields | Observer 1 (μm) | Observer 2 (μm) | ICC (95% CI) | MD (μm) | SW (μm) | 95% LOA LB (μm) | 95% LOA UB (μm) | CR (μm) |
| --- | --- | --- | --- | --- | --- | --- | --- | --- |
| Central | 296.1 ± 97.2 | 297.1 ± 97.5 | 0.994 (0.993-0.995) | 0.99 ± 10.60 | 7.49 | -19.78 | 21.76 | 20.77 |
| Inner superior | 291.5 ± 92.8 | 293.3 ± 93.6 | 0.994 (0.993-0.995) | 1.77 ± 9.88 | 6.98 | -17.59 | 21.13 | 19.36 |
| Inner temporal | 283.5 ± 92.2 | 286.0 ± 92.9 | 0.992 (0.990-0.994) | 2.48 ± 11.51 | 8.14 | -20.08 | 25.05 | 22.57 |
| Inner inferior | 284.7 ± 94.7 | 285.5 ± 94.9 | 0.992 (0.990-0.993) | 0.80 ± 12.31 | 8.70 | -23.32 | 24.92 | 24.12 |
| Inner nasal | 279.3 ± 96.1 | 279.7 ± 97.1 | 0.995 (0.994-0.996) | 0.38 ± 9.60 | 6.78 | -18.43 | 19.18 | 18.81 |
| Outer superior | 281.0 ± 81.3 | 281.5 ± 81.9 | 0.988 (0.986-0.991) | 0.56 ± 12.39 | 8.76 | -23.72 | 24.83 | 24.28 |
| Outer temporal | 263.3 ± 80.8 | 265.0 ± 81.3 | 0.993 (0.991-0.994) | 1.74 ± 9.57 | 6.77 | -17.01 | 20.49 | 18.75 |
| Outer inferior | 265.0 ± 88.2 | 266.1 ± 87.2 | 0.990 (0.988-0.992) | 1.10 ± 12.34 | 8.73 | -23.09 | 25.29 | 24.19 |
| Outer nasal | 225.6 ± 82.3 | 226.7 ± 83.9 | 0.994 (0.993-0.995) | 1.05 ± 8.91 | 6.30 | -16.42 | 18.52 | 17.47 |

ICC = intraclass correlation coefficient; CI = confidence interval; MD = mean difference between two observers; SW = within subject standard deviation; LOA LB= limit of agreement lower boundary; LOA UB = limit of agreement upper boundary; CR = coefficient of reproducibility
